# Valine improves mitochondrial function and protects against oxidative stress

**DOI:** 10.1093/bbb/zbad169

**Published:** 2023-12-13

**Authors:** Shakshi Sharma, Xiaomin Zhang, Gohar Azhar, Pankaj Patyal, Ambika Verma, Grishma KC, Jeanne Y Wei

**Affiliations:** Donald W. Reynolds Department of Geriatrics, Institute on Aging, University of Arkansas for Medical Sciences, Little Rock, AR, USA; Donald W. Reynolds Department of Geriatrics, Institute on Aging, University of Arkansas for Medical Sciences, Little Rock, AR, USA; Donald W. Reynolds Department of Geriatrics, Institute on Aging, University of Arkansas for Medical Sciences, Little Rock, AR, USA; Donald W. Reynolds Department of Geriatrics, Institute on Aging, University of Arkansas for Medical Sciences, Little Rock, AR, USA; Donald W. Reynolds Department of Geriatrics, Institute on Aging, University of Arkansas for Medical Sciences, Little Rock, AR, USA; Donald W. Reynolds Department of Geriatrics, Institute on Aging, University of Arkansas for Medical Sciences, Little Rock, AR, USA; Donald W. Reynolds Department of Geriatrics, Institute on Aging, University of Arkansas for Medical Sciences, Little Rock, AR, USA

**Keywords:** valine, mitochondrial function, oxidative stress, reactive oxygen species

## Abstract

Among the branched-chain amino acids, leucine and isoleucine have been well studied for their roles in improving mitochondrial function and reducing oxidative stress. However, role of valine in mitochondrial function regulation and oxidative stress management remains elusive. This study investigated valine effect on mitochondrial function and oxidative stress *in vitro*. Valine increased expression of genes involved in mitochondrial biogenesis and dynamics. It upregulates mitochondrial function at complexes I, II, and IV levels of electron transport chain. Flow cytometry studies revealed, valine reduced oxidative stress by significantly lowering mitochondrial reactive oxygen species and protein expression of 4-hydroxynonenal. Functional role of valine against oxidative stress was analyzed by XFe96 Analyzer. Valine sustained oxidative phosphorylation and improved ATP generation rates during oxidative stress. In conclusion, our findings shed more light on the critical function of valine in protecting mitochondrial function thereby preventing mitochondrial/cellular damage induced by oxidative stress.

## Graphical Abstract

**Graphical Abstract:**
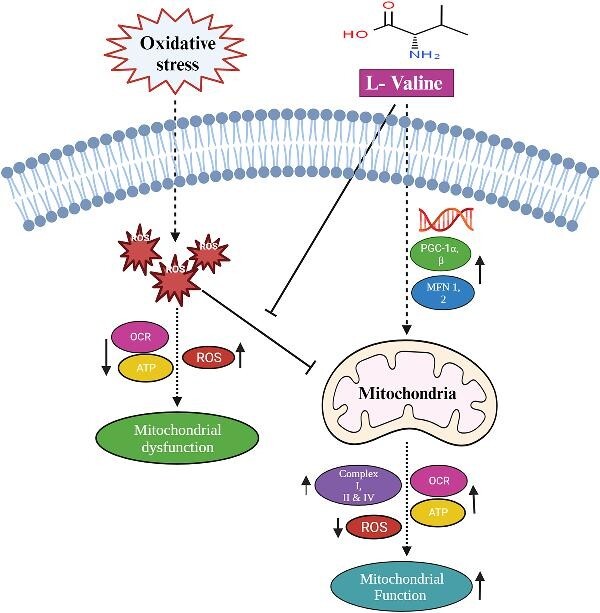
Valine improves mitochondrial function and protects against oxidative stress. It modulates oxidative phosphorylation, ATP production, and inhibits mitochondrial dysfunction by reducing reactive oxygen species (ROS) production.

Branched-chain amino acids (BCAAs) are among the most prevalent essential amino acids that are pivotal for the health of the human body (Nie *et al.*^2018^). Leucine, isoleucine, and valine are α-amino acids with aliphatic side chains that are important for maintaining the body's nitrogen balance (Doi *et al.*^2003^) as well as skeletal muscle cell growth and development and have been linked to multiple metabolic processes, that are important in cellular pathways, and are central in the relationship between diet, health, and aging (Wolfe 2017; Green and Lamming 2019; Vanweert *et al.*^2022^). The activation of pathways including mammalian target of rapamycin, phosphoinositide 3-kinase-protein kinase B (PI3K-AKT), and maintenance of physiological metabolic health are all influenced by these amino acids (Viana *et al.*^2019^; Mann *et al.*^2021^). Aside from helping with protein synthesis, they also aid in translation and degradation (Tom and Nair 2006). The potential of BCAAs as dietary supplements for treating various disorders has been studied in both human and mouse models and has been shown to influence metabolic activity, improve mitochondrial bioenergetics, support muscle growth, and maintain mitochondrial health (Lynch and Adams 2014; Holecek 2018; Ruocco *et al*. 2021). Mitochondria are crucial for producing adenosine triphoshate (ATP) and also participate in fatty acid oxidation, cell survival, and apoptosis (Hewton *et al.*^2021^; Yoneshiro *et al.*^2021^). The activation of multiple genes that regulate mitochondrial function, including peroxisome proliferator-activated receptor gamma (PPAR-γ) coactivator-1 alpha (PGC-1α), PPAR-gamma coactivator-1 beta (PGC-1β), and the mitofusins, by BCAAs, in turn stimulates mitochondrial biogenesis. According to several studies (Hinkle *et al.*^2022^), mitochondrial dysfunction is associated with oxidative stress, cellular damage and is thought to be a common underlying mechanism of metabolic diseases (Rocha *et al.*^2013^; Walters *et al.*^2016^). Extreme reactive oxygen species (ROS) production can hinder mitochondrial membrane permeability, and produce lipid peroxidation products, which leads to mitochondrial-related apoptosis (Ren *et al.*^2017^; Zhao *et al.*^2019^). In skeletal muscle, ROS generation induces a signaling cascade that can produce muscle damage (Lee *et al.*^2017^; Pan and Chen 2021). It has been demonstrated that adding BCAA supplements lengthens the average lifespan of mice by boosting the ROS defense mechanism in the skeletal and cardiac muscle tissues and improving mitochondrial biogenesis (Valerio *et al.*^2011^). BCAAs alone or together can mediate the antioxidant defense system and reduce oxidative stress in skeletal muscles in middle-aged mice (D'Antona *et al.*^2010^; Cruzat *et al.*^2014^). Valine (Val), a necessary BCAA, can also act as an energetic molecule and is important for protein synthesis and growth of skeletal muscle (Kohlmeier 2003). Further, valine also plays a favorable function in lipid metabolism (Bishop *et al.*^2020^) and have inflammatory properties (Gart *et al.*^2022^).

While studies have been conducted on BCAAs, especially leucine and isoleucine on mitochondrial function, cellular metabolism and oxidative stress (Mattick *et al.*^2013^; Tamanna and Mahmood 2014), there is a dearth of literature on the cellular and functional importance of valine. In the present study, we evaluated the effect of valine treatment on mitochondrial function and its response to oxidative stress. Our findings demonstrate that valine administration improved oxygen consumption rate (OCR) and overall ATP generation in the mitochondria. Additionally, valine treatment also enhanced the transcriptional activity of PGC-1α, PGC-1β, and other genes that regulate mitochondrial function. Valine significantly reduced ROS production, thereby maintaining the OCR during H_2_O_2_-mediated oxidative stress. The findings from this study enhance our understanding of valine as an important nutrient to improve mitochondrial function, thereby protecting cells against oxidative damage.

## Materials and methods

### Cell culture and cell proliferation assay

The C2C12 mouse cell line used in this study was purchased from the American Type Culture Collection (ATCC, CRL-1772). Cell culture reagents were obtained from Thermo Fischer Scientific as previously described (Rogers *et al.*^2013^). Valine was obtained from Sigma–Aldrich, St. Louis, MO, USA, (V0500), and used following the manufacturer's protocol. C2C12 cell viability after valine treatment was determined by using MTS reagent (3-(4,5-dimethylthiazol-2-yl)-5-(3-carboxymethoxyphenyl)-2-(4-sulfophenyl)-2H-tetrazolium) (Abcam; ab197010). A 200 µL C2C12 cell suspension (5 × 10^3^/well) was seeded in a 96-well plate. At 70%-80% confluency, cells were treated with two different concentrations (1.0 and 1.5 m m) of valine for 24 h. A volume of 20 µL/well MTS reagent was added in each well and incubated for a period of 0.5-4 h at 37°C following the manufactures’ protocol. The absorbance was recorded at 490 nm.

### Measurement of ATP and NAD/NADH levels

C2C12 cells (5 × 10^3^ cells/well) were plated in 96-well plate, treated with 1.0 m m concentration of valine for 24 h. Intracellular ATP levels were assessed using the CellTiter-Glo 2.0 assay kit (Promega, Madison, WI, USA; G9242). Further, the NAD/NADH (nicotinamide adenine dinucleotide) levels after valine treatment were determined using a NAD/NADH Glo Assay Kit (Promega, Madison, WI, USA; G9071) following the manufacturer's protocol.

### RNA isolation and quantitative reverse-transcriptase PCR

Cells were pre-treated with 1.0 m m concentration of valine for 24 h. Total cellular RNA was extracted from cells utilizing the RNeasy Kit (Qiagen) in accordance with the manufacturer's protocol. Total RNA obtained was then revere transcribed to Complementary DNA (cDNA) using cDNA Reverse Transcription Kit (Applied Biosystems, Thermo-Fischer Scientific, Waltham, MA, USA; 4368814). The real-time quantitative reverse transcription polymerase chain reaction (qRT-PCR) amplification was performed using SYBR Green master mix (Applied Biosystems, Thermo Fischer Scientific, Waltham, MA, USA; 4344463) in a QuantStudio^TM^ 3 Real-Time PCR instrument (Applied Biosystems). The relative expression was measured using StepOne software. All experiments were performed as described previously (Rogers *et al.*^2013^). Primer set used for qPCR were listed in Table S1.

### Western blot analysis

C2C12 cells were cultured and treated with valine as mentioned above and protein concentration was determined by using a BCA protein assay kit (Thermo–Fisher, Waltham, MA, USA). Western blotting was conducted as previously described (Zhang *et al.*^2012^). The antibodies used in the study were PGC-1α (sc-518025) and anti-4HNE antibody (ab46545), anti-mouse HRP (Invitrogen, Carlsbad, CA, USA; 62-6520) and anti-rabbit AP (Bio-Rad, Hercules, CA, USA; 64251130). iBright™ CL1500 (Invitrogen) was used to visualized the protein signal and ImageJ software v1.53t (National Institutes of Health, Bethesda, MD, USA) was used for quantification of protein expression.

### Extracellular flux analysis

C2C12 cells were seeded in XFe96 Well plates at a density of 1.2 × 10^4^ cells/well (Seahorse Bioscience, Billerica, MA, USA). The cells were then treated with 1.0 m m concentration of valine for 24 h respectively. After valine treatment, Seahorse XFe96 Extracellular Flux Analyzer was used to measure OCR using XF Cell Mito Stress Test Kit (Agilent, Santa Clara, CA, USA; 103015-100) and extracellular acidification rate (ECAR) using XF Glycolytic Rate Assay Kit (Agilent, Santa Clara, CA, USA; 103344-100). For determination of ATP production rate from mitochondria and glycolysis, XF-Real-time ATP Assay Kit (Agilent, Santa Clara, CA, USA; 103592-100). All metabolic assays were performed following the procedure from the manufacturer.

### High-resolution respirometry

The mitochondrial respiration in the intact C2C12 cells (1 × 10^6^/sample) after valine treatment was determined using Oxygraph-O2K high-resolution respirometer (Oroboros Instruments GmbH, Innsbruck, Austria) (Raiteri *et al.*^2021^). The mitochondrial respiratory activity at different complexes was analyzed using substrate-uncoupler-inhibitor-titration protocol as described in (Patyal *et al.*^2022^). The OCR from all mitochondrial complex was expressed as oxygen flux (pmol/s*Million Cells). DatLab 6.2 software (Innsbruck, Austria) was used to perform the data analysis.

### Oxidative stress analysis

C2C12 cells were seeded at a density of 5 × 10^3^ cells/mL (200 µL) in 96-well plate. H_2_O_2_ was obtained from Sigma–Aldrich, St. Louis, MO, USA (88 597). A volume of 200-1000 µmol/L treatment of H_2_O_2_ generates oxidative stress in C2C12 cells as previously reported (Li *et al.*^2020^). Cells were grown overnight and next day exposed to different concentrations of H_2_O_2_ (200, 400, 600, and 800 µmol/L) to stimulate oxidative stress for 6 h (Wu *et al.*^2022^). A total of 400 µmol/L concentration of H_2_O_2_ was selected as the final concentration for treatment. After H_2_O_2_ treatment, cells were treated with 1.0 m m concentration of valine for 24 h to assess cell proliferation viability as described above. Four groups used in this and subsequent experiments were control group (without any treatment), valine treated group, H_2_O_2_ treated group, H_2_O_2_ + valine treatment group.

### Flow cytometry analysis

MitoSOXTM Red (Thermo Fisher, Eugene, OR, USA; M3600) was used to detect the mitochondrial ROS production. The C2C12 cells (1 × 10^6^ cells/well), were plated in 6-well culture plate and treated with 400 µmol/L H_2_O_2_ for 6 h. Following H_2_O_2_ treatment, cells were treated with a final concentration of 1.0 m m of valine for 24 h. After 24 h, cells were incubated with 5 µm of MitoSox Red for 10 min at 37 °C as previously described (Verma *et al.*^2023^). Flow cytometer (BD LSRFortessaTM Cell Analyzer, Franklin Lakes, NJ, USA) was used to quantify the fluorescence intensity and data were processed by FlowJo_v10.8.1 software.

### Statistical analysis

Results obtained from this study were described as mean ± standard deviation. The 2-tailed Student's *t-*test was used to determine the statistical significance between two groups. Multiple groups were ascertained by using one-way analysis of variance (ANOVA) followed Tukey's multiple comparison test. All the *P* values < 0.05 were considered to indicate as statistically significant. GraphPad Prism 9.11 Software Inc. (Dotmatics, Boston, MA, USA) was utilized to process the statistical analysis.

## Results

### Valine increases ATP and NAD/NADH production

Valine treatment at two different concentrations (1.0 and 1.5 m m) for 24 h, showed no significant effect on C2C12 cells viability as compared to control as shown in Figure S1. We next investigated the effect of valine treatment on ATP and NAD/NADH production rate. The treatment of valine at 1.0 m m concentration for 24 h, resulted in significantly higher ATP and NAD/NADH production rates (Figure S2a and b). Therefore, we selected this concentration for further experiments.

### Valine impacts genes that regulate mitochondrial biogenesis and function

**Figure 1.**
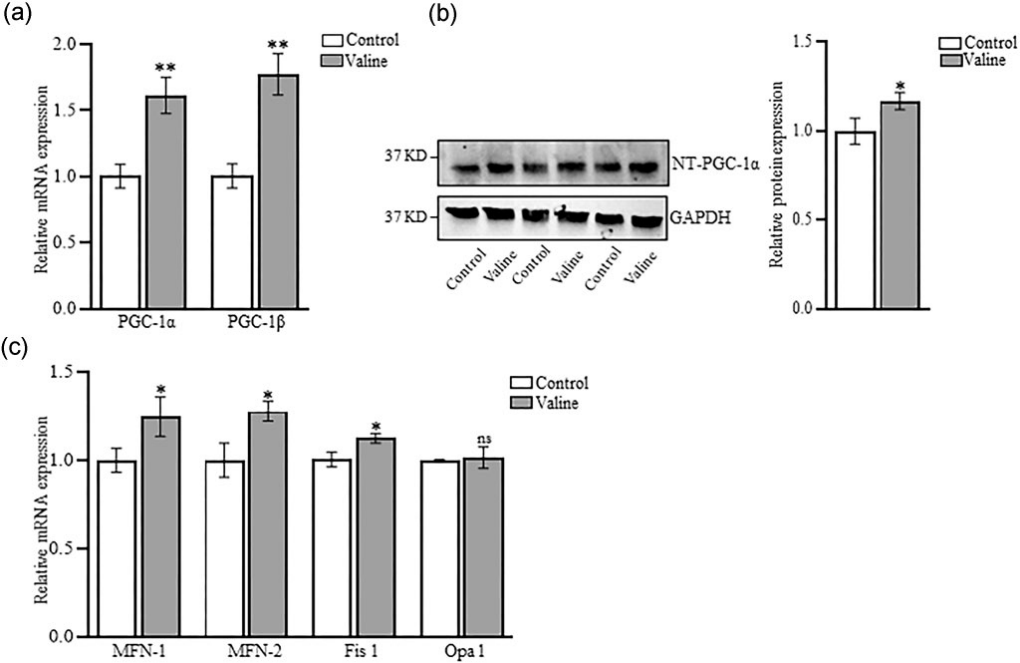
Valine treatment upregulated expression of important mitochondrial genes in C2C12 cells (a) The mRNA expression levels of *PGC1-α* and *PGC1-β* were determined using Real-time RT-qPCR (b) The protein levels of PGC-1α was determined using western blotting analysis with gleyecelarldehyde 3-phosphate dehydrogenase (GAPDH) being used as loading control (c) Gene expression levels of *MFN1, MFN2*, and *Fis1* were increased with no change in *Opa1* levels (*n* = 3). *P < 0.05, **P < 0.01, ns: *P* > 0.05.

The mRNA expression levels of PGC-1α and PGC-1β (Figure 1a) were upregulated following valine treatment. The protein expression of PGC-1α was also found to be significantly higher in the valine-treated cells (Figure 1b). Further, valine treatment also upregulated the expression levels of mitofusin-1 (MFN1), mitofusin-2 (MFN2), and mitochondrial fission 1 (Fis1), whereas no difference was observed on optic atrophy-1 (Opa 1) gene expression (Figure 1c).

### Valine distinctively regulates mitochondrial bioenergetics

**Figure 2.**
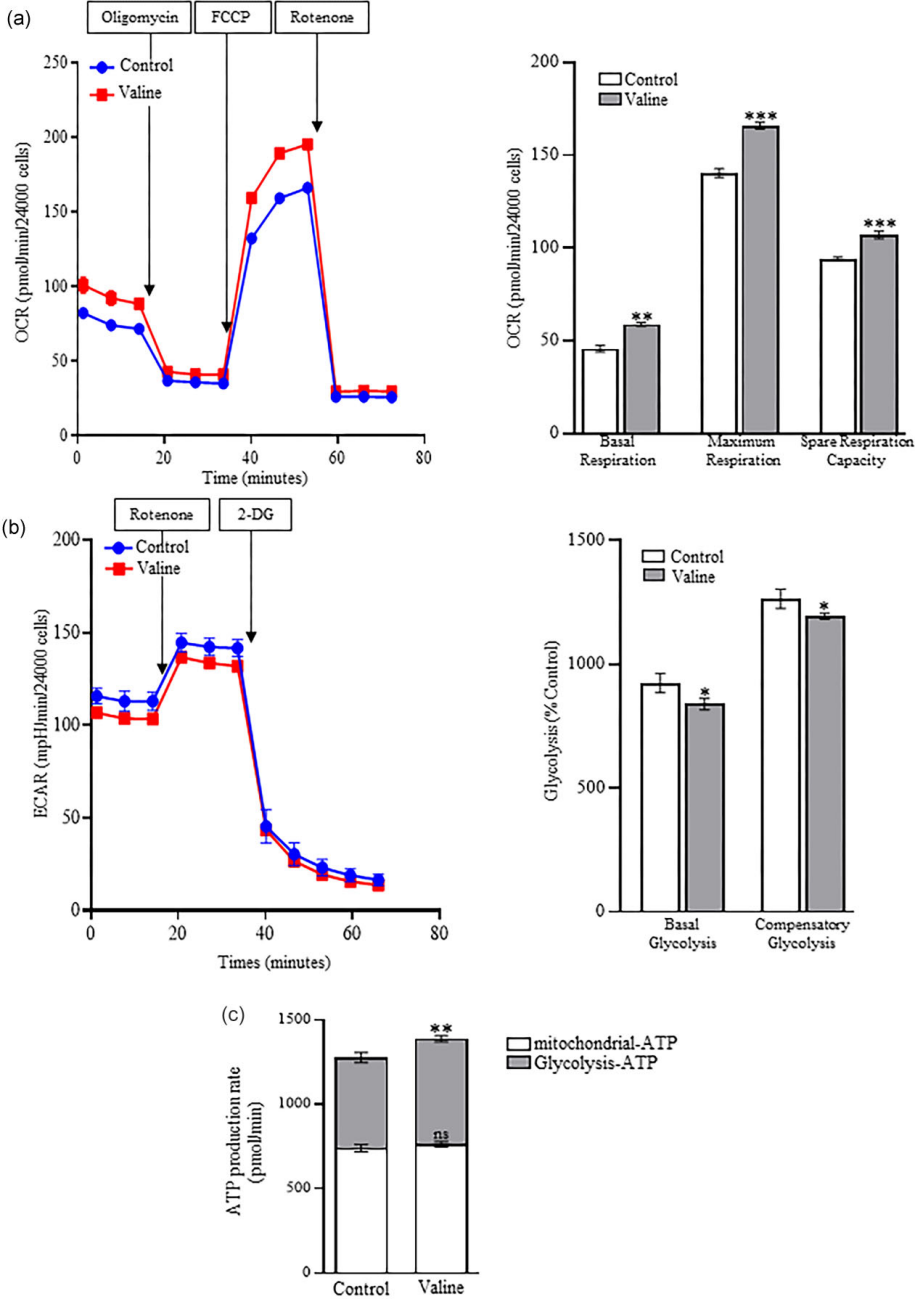
Valine treatment increased OCR and total ATP and reduced glycolytic rate (ECAR) in C2C12 cells. (a) Valine upregulated the basal respiration, maximum respiration, and spare respiratory capacity in C2C12 cells. (b) In ECAR, both basal and compensatory glycolysis were significantly reduced following treatment. (c) Total ATP production was significantly upregulated in oxidative phosphorylation (mitochondrial-ATP) but showed no change in glycolysis (glycolysis-ATP) (*n* = 4). *P < 0.05, **P < 0.01, ***P < 0.001, ns: *P* > 0.05.

Valine treatment resulted in increased OCR production and, therefore improved the mitochondrial function in C2C12 cells. Valine treatment elevated all of the different domains of OCR such as basal respiration, maximal respiratory capacity, and spare respiratory capacity (Figure 2a). ECAR analysis resulted in reduced basal glycolysis and compensatory glycolysis following valine treatment (Figure 2b). However, valine treatment significantly increased the mitochondrial ATP production rate, due to an increase in the oxidative phosphorylation (OXPHOS) activity (Figure 2c).

### Valine specifically regulates the mitochondrial respiratory chain complex levels

**Figure 3.**
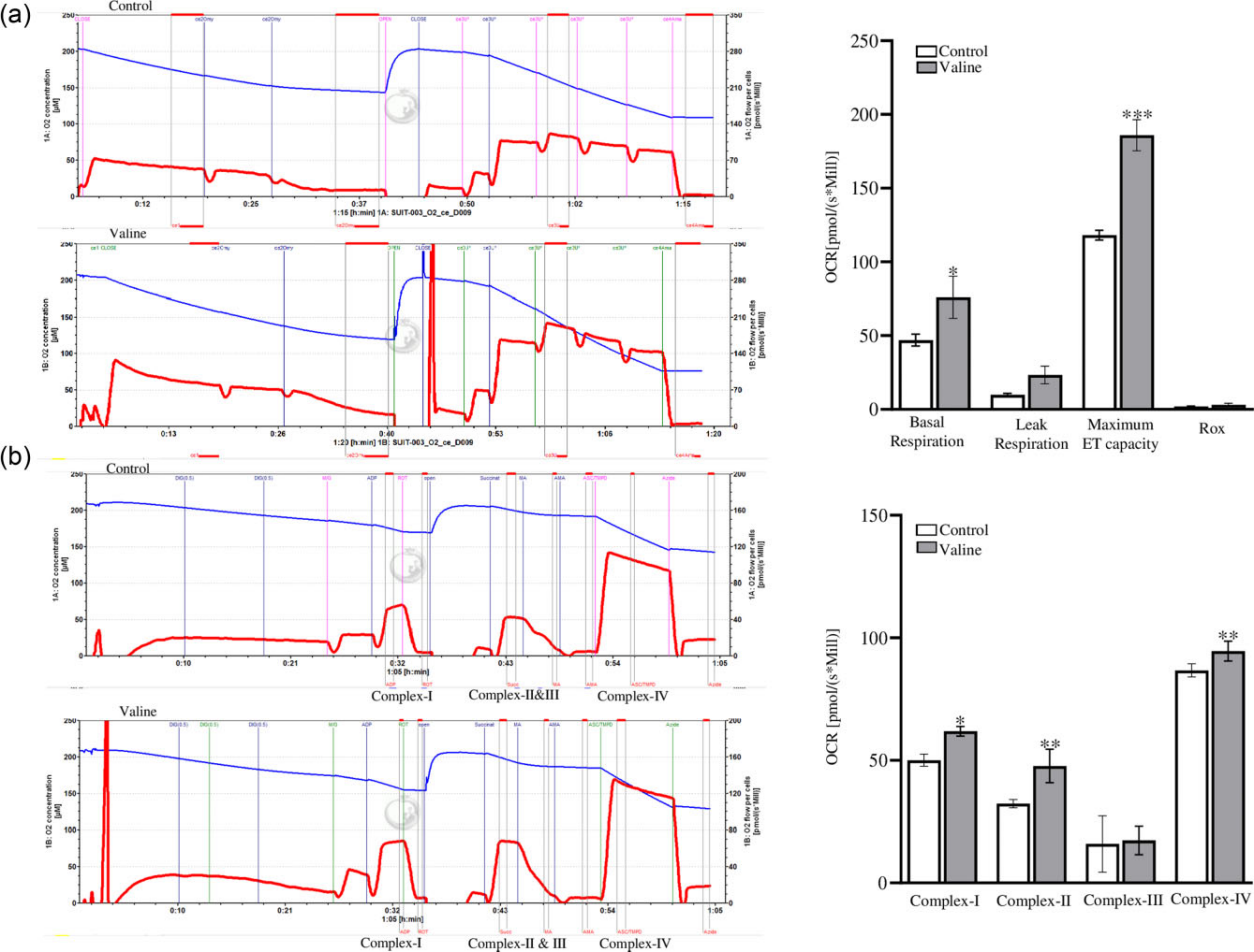
High-resolution respiratory analysis showed an increased mitochondrial respiration following valine treatment. (a) C2C12 cells were treated with 0.1 m m concentration of valine for 24 h. Basal respiration and maximum ET capacity were increased following the treatment (b) Measurement of OCR at different complexes of ETC showed increase in complexes I, II, and IV activity after treatment (*n* = 3). * *P* < 0.05, **P < 0.01, ***P < 0.001, ns*: P* > 0.05.

Oxygraph-2 K (O2K) was used to measure the mitochondrial function by analyzing the high-resolution respiratory capacity in C2C12 cells. Valine treatment significantly increased the basal respiration and the maximal capacity of mitochondrial electron transport chain (ETC) (Figure 3a) in C2C12 cells. We then further assessed the effect of valine treatment on different complexes of the ETC. The respiration rates of complexes I (NADH/ubiquinone oxidoreductase), II (succinate dehydrogenase), and IV (cytochrome c oxidase) increased after treatment. No change was observed in complex III activity (cytochrome c reductase) (Figure 3b).

### Valine reduced mitochondrial ROS production induced by H_2_O_2_

**Figure 4.**
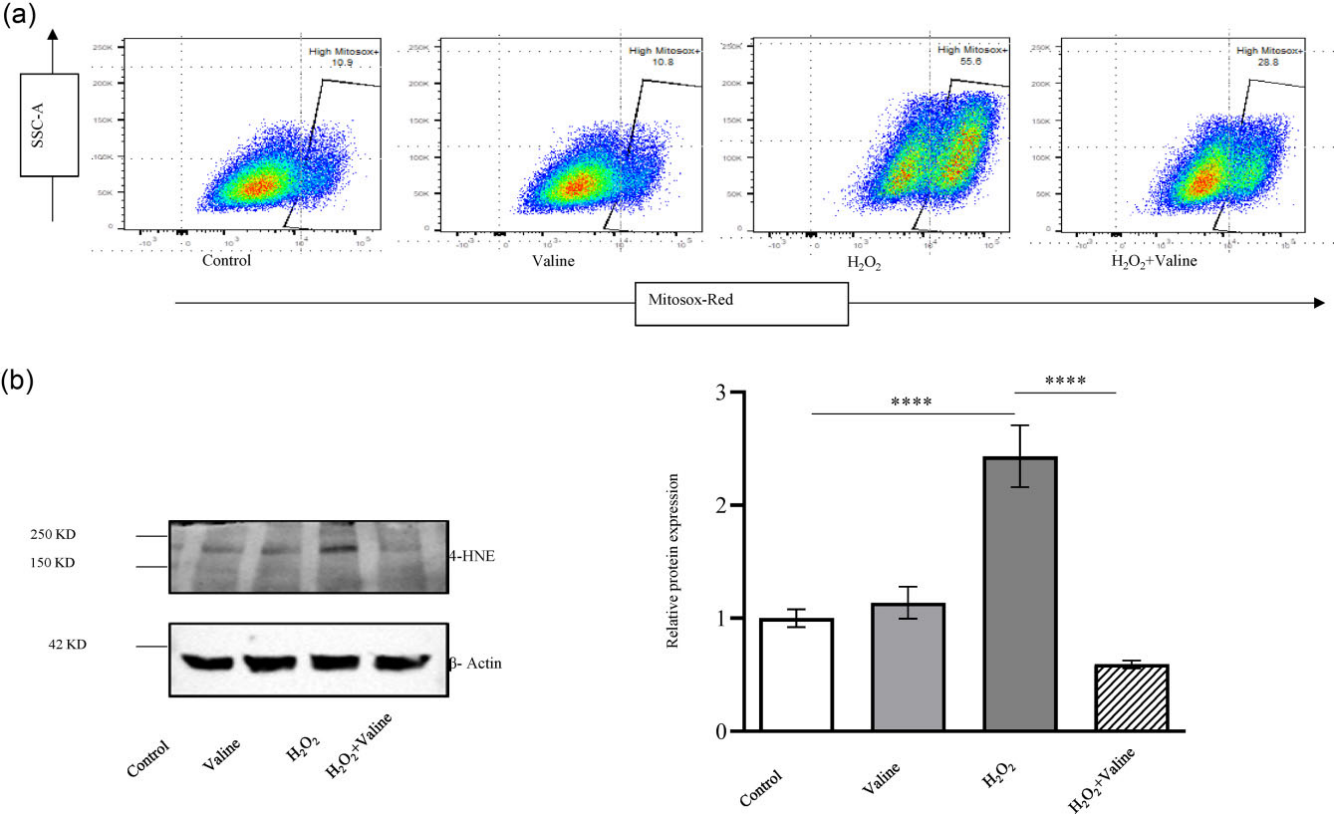
Valine reduced mitochondrial ROS production in C2C12 cells. (a) ROS generation was evaluated using a flow cytometer. H_2_O_2_ treatment caused ROS production in 55.6% of cells, whereas, when H_2_O_2_ was used along with valine, recovery from oxidative stress was improved and only 28.8% of cells were ROS positive. (b) The protein expression of 4-HNE antibody was assessed by western blotting technique with β-actin as a loading control (*n* = 3). ****P < 0.0001.

We first examined the effect of H_2_O_2_ treatment on the cellular viability of C2C12 cells. The results showed that treatment of H_2_O_2_ at different concentrations for 6 h affected cell viability in dose-dependent manner (Figure S3). We selected 400 µmol/L concentration of H_2_O_2_ as our final treatment, as the recovery rate at this dose was found to be higher after valine treatment as compared to the other higher concentration used (Figure S4). The results showed that valine treatment significantly recovered the cell viability in C2C12 cells affected by H_2_O_2_ treatment (Figure S5). Further, we observed that H_2_O_2_ treatment significantly upregulate the ROS production. Our results showed that treatment of valine, along with H_2_O_2_, prevented ROS production (Figure 4a). In addition, H_2_O_2_ treatment also increased the protein expression level of 4-hydroxynonenal (4-HNE), which was prevented by valine treatment.

### Valine protects against oxidative stress and metabolic alterations

**Figure 5.**
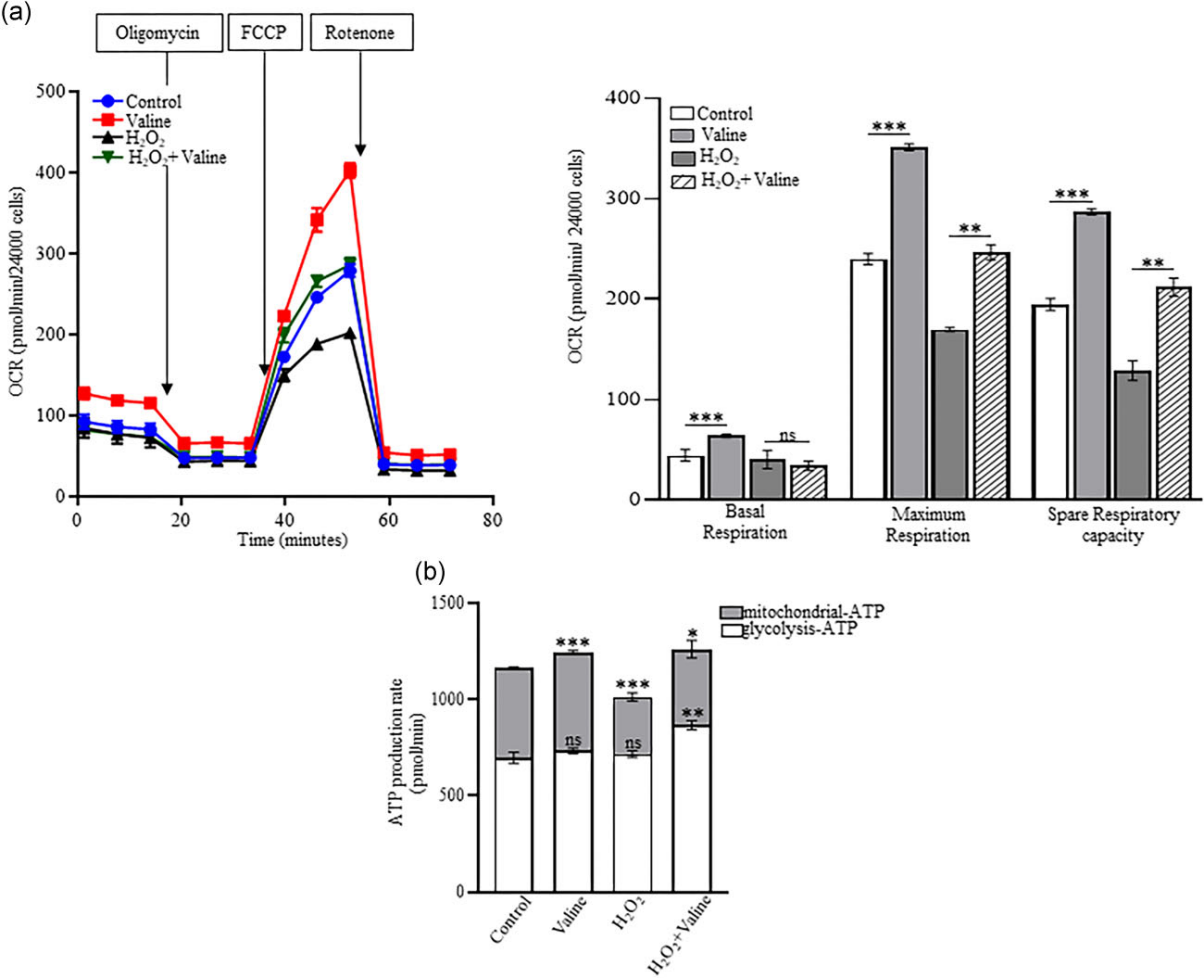
Protective role of valine against oxidative stress. (a) OCR, Maximum respiration and spare respiratory capacity was repressed due to oxidative stress after H_2_O_2_ treatment, which was restored after valine treatment. (b) Total ATP production was decreased in both oxidative phosphorylation and glycolysis, which was recovered after valine treatment (*n =* 4). *P < 0.05, **P < 0.01, ***P < 0.001, **** *P* < 0.0001, ns: *P* > 0.05.

To confirm the protective role of valine against oxidative stress, we next assessed its impact on oxidative phosphorylation. Data were normalized to remove variation due to cell density before the experiment. H_2_O_2_ treatment initially repressed basal respiration, maximal respiratory capacity and spare respiratory capacity (Figure 5a). Valine treatment alone and in the presence of H_2_O_2_ significantly improved the maximal respiratory capacity and spare respiratory capacity. Additionally, we also determined the effect of H_2_O_2_ on total ATP production rate. H_2_O_2_ treatment significantly reduced the mitochondrial ATP rate, which was restored and improved by valine treatment (Figure 5b).

## Discussion

This study has three major findings: first, valine plays a crucial role in improvement of cellular mitochondrial function; second, valine has a protective effect against oxidative stress by minimizing the production of mitochondrial ROS; and third, valine maintains oxidative phosphorylation and ATP rate during oxidative stress. Clinically, BCAAs intake have been suggested to reduce sarcopenia in older adults and possibly improve glucose metabolism, potentially by increasing mitochondrial biogenesis and muscle cellular function (Yoon 2016; Kitajima *et al.*^2018^). Despite many studies demonstrating the correlation between BCAAs and insulin resistance (Schnuck *et al.*^2016^), valine was reported not to cause insulin sensitivity or worsen insulin resistance (Rivera *et al.*^2020^; Mao *et al.*^2022^). We are among the first to report on the key role of the BCAA, valine, in enhancing mitochondrial function and reducing oxidative stress in a skeletal muscle cell line. Our study focused on exploring whether valine could be utilized to improve mitochondrial function. We analyzed the effect of valine treatment on cell viability of C2C12 cells. Valine treatment showed no change in cell viability. Our observation is consistent with no effect of varying concentrations (0.5 m m–2 m m) of valine for up to 48 h of treatment in C2C12 cells (Rivera *et al.*^2020^).

Our study suggests that mitochondrial biogenesis is improved with valine treatment. PGC-1α and PGC-1β are the main transcriptional coactivators of oxidative phosphorylation in mitochondria and also regulate mitochondrial biogenesis and function (Kang and Li 2012). Other studies have shown that leucine stimulates expression of PGC-1α and PGC-1β to promote mitochondrial biogenesis (Liang *et al.*^2014^; Johnson *et al.*^2018^; Rivera *et al.*^2020^; Ye *et al.*^2020^). Similarly, we found that valine regulates mitochondrial biogenesis and dynamics by increasing the expression levels of PGC-1α, PGC-1β, MFN1, MFN2, and Fis1. Mitochondria play a predominant role in regulating energy metabolism, ATP production and oxygen consumption and for the maintenance of cellular functions (Chu *et al.*^2021^). The increased OCR is an important indicator, which reflects the mitochondrial respiratory chain complex activity (du Plessis *et al.*^2015^). Our data showed increased OCR and reduced ECAR in C2C12 cells following valine treatment. This reduction in ECAR could be due to extreme oxidation of fatty acids, which inactivate pyruvate dehydrogenase and obstruct the normal process of glycolysis (Savage *et al.*^2007^). Others have reported that valine did not alter mitochondrial biogenesis or glycolysis (Rivera *et al.*^2020^). We also observed an increase in total ATP production in C2C12 cells following valine treatment, which emphasized the crucial role mitochondrial oxidative phosphorylation plays in sustaining energy status (Zheng 2012). Interestingly, our findings highlighted the important part that valine plays in improving mitochondrial activity at electron transport complex levels, including complex I, II, and IV. Recent studies have focused on inhibiting Complex I to target the Warburg effect and metabolic plasticity of cancer cells (Chaube *et al.*^2015^). The role of valine in regulating complex I is well demonstrated in T-cell acute lymphoblastic leukemia (Thandapani *et al.*^2022^). It is possible that valine could act as an anaplerotic metabolite and drive the tricarboxylic acid cycle to sustain mitochondrial ATP production.

Muscle tissues are most vulnerable to a high risk of oxidative stress (Li *et al.*^2020^). Oxidative stress can impair mitochondrial oxidation and worsen metabolic disorders (Lian *et al.*^2022^). Extreme levels of ROS production can impair skeletal muscle function by harming the cells through oxidation (Zhang *et al.*^2001^; Zhao *et al.*^2021^). Our results indicate that valine significantly reduced mitochondrial ROS production and protein expression of 4-HNE in C2C12 cells, and thus can help in promoting the growth of muscle and the healing of damaged tissue. Interestingly, valine also protects the cell by sustaining oxidative phosphorylation and generating more ATP for energy supplementation to counter the H_2_O_2_-induced oxidative stress (Jeong *et al.*^2022^). In accordance with our findings, cysteine was reported to reduce the H_2_O_2_-induced mitochondrial stress and restore mitochondrial function in C2C12 myoblasts (Mizugaki *et al.*^2022^). Valine could potentially be used therapeutically to support good mitochondrial activity and assist in reducing cellular and mitochondrial damage induced by ROS-driven oxidative stress.

### Limitations

This study primarily focused on determining the effect of valine treatment on one cell line. Further research is required to completely delineate the mechanism (s) by which valine might help in cell survival during stress in different cell lines and *in vivo.*

## Conclusion

In conclusion, we found that valine treatment does improve mitochondrial function, by enhancing mitochondrial oxidative phosphorylation and total ATP production. This could be due to its role in the improvement of the gene expression of PGC-1α, PGC-1β, and those involved in biogenesis and function. By lowering the H_2_O_2_-driven oxidative stress, valine apparently preserves cell viability, maximizes respiratory capacity, and lowers the formation of mitochondrial ROS. The findings of this study provide insight into valine's role in providing mitochondrial protection against oxidative stress, and suggest that it may be a future target for maintaining cellular function under stress.

## Supplementary Material

zbad169_Supplemental_File

## Data Availability

The dataset generated during and/ or analyzed during the current study are available from the corresponding author on reasonable request.

## References

[bib1] Bishop CA , Schulze MB, Klaus S et al. The branched-chain amino acids valine and leucine have differential effects on hepatic lipid metabolism. FASEB J 2020;34:9727-39. 32506644 10.1096/fj.202000195R

[bib2] Chaube B , Malvi P, Singh SV et al. Targeting metabolic flexibility by simultaneously inhibiting respiratory complex I and lactate generation retards melanoma progression. Oncotarget 2015;6:37281-99. 26484566 10.18632/oncotarget.6134 PMC4741930

[bib3] Chu YD , Lim SN, Yeh CT et al. COX5B-mediated bioenergetic alterations modulate cell growth and anti-cancer drug susceptibility by orchestrating claudin-2 expression in colorectal cancers. Biomedicines 2021;10:60. 35052740 10.3390/biomedicines10010060 PMC8772867

[bib4] Cruzat VF , Krause M, Newsholme P. Amino acid supplementation and impact on immune function in the context of exercise. J Int Soc Sports Nutr 2014;11:61. 25530736 10.1186/s12970-014-0061-8 PMC4272512

[bib5] D'Antona G , Ragni M, Cardile A et al. Branched-chain amino acid supplementation promotes survival and supports cardiac and skeletal muscle mitochondrial biogenesis in middle-aged mice. Cell Metab 2010;12:362-72. 20889128 10.1016/j.cmet.2010.08.016

[bib6] Doi M , Yamaoka I, Fukunaga T et al. Isoleucine, a potent plasma glucose-lowering amino acid, stimulates glucose uptake in C2C12 myotubes. Biochem Biophys Res Commun 2003;312:1111-7. 14651987 10.1016/j.bbrc.2003.11.039

[bib7] du Plessis S , Agarwal A, Mohanty G et al. Oxidative phosphorylation versus glycolysis: what fuel do spermatozoa use? Asian J Androl 2015;17:230. 25475660 10.4103/1008-682X.135123 PMC4650467

[bib8] Gart E , van Duyvenvoorde W, Caspers MPM et al. Intervention with isoleucine or valine corrects hyperinsulinemia and reduces intrahepatic diacylglycerols, liver steatosis, and inflammation in ldlr-/-.Leiden mice with manifest obesity-associated NASH. FASEB J 2022;36:e22435. 35830259 10.1096/fj.202200111R PMC12166278

[bib9] Green CL , Lamming DW. Regulation of metabolic health by essential dietary amino acids. Mech Ageing Dev 2019;177:186-200. 30044947 10.1016/j.mad.2018.07.004 PMC6333505

[bib10] Hewton KG , Johal AS, Parker SJ. Transporters at the interface between cytosolic and mitochondrial amino acid metabolism. Metabolites 2021;11:112. 33669382 10.3390/metabo11020112 PMC7920303

[bib11] Hinkle JS , Rivera CN, Vaughan RA. Branched-chain amino acids and mitochondrial biogenesis: an overview and mechanistic summary. Mol Nut Food Res 2022;66:2200109. 10.1002/mnfr.202200109 PMC9786258 36047448

[bib12] Holeček M. Branched-chain amino acids in health and disease: metabolism, alterations in blood plasma, and as supplements. Nutr Metab (Lond) 2018;15:33. 29755574 10.1186/s12986-018-0271-1 PMC5934885

[bib13] Jeong MJ , Lim DS, Kim SO et al. Protection of oxidative stress-induced DNA damage and apoptosis by rosmarinic acid in murine myoblast C2C12 cells. Biotechnol Bioproc E 2022;27:171-82.

[bib14] Johnson MA , Gannon NP, Schnuck JK et al. Leucine, palmitate, or leucine/palmitate cotreatment enhances myotube lipid content and oxidative preference. Lipids 2018;53:1043-57. 30706482 10.1002/lipd.12126

[bib15] Kang C , Li Ji L. Role of PGC-1α signaling in skeletal muscle health and disease. Ann NY Acad Sci 2012;1271:110-7. 23050972 10.1111/j.1749-6632.2012.06738.x PMC3499658

[bib16] Kitajima Y , Takahashi H, Akiyama T et al. Supplementation with branched-chain amino acids ameliorates hypoalbuminemia, prevents sarcopenia, and reduces fat accumulation in the skeletal muscles of patients with liver cirrhosis. J Gastroenterol 2018;53:427-37. 28741271 10.1007/s00535-017-1370-x

[bib17] Kohlmeier M. Valine. In: Nutrient Metabolism. Cambridge, Massachusetts, USA: Elsevier, 2003: 370-7.

[bib18] Lee MH , Han MH, Lee DS et al. Morin exerts cytoprotective effects against oxidative stress in C2C12 myoblasts via the upregulation of Nrf2-dependent HO-1 expression and the activation of the ERK pathway. Int J Mol Med 2017;39:399-406. 28035409 10.3892/ijmm.2016.2837

[bib19] Li J , Yang Q, Han L et al. C2C12 mouse myoblasts damage induced by oxidative stress is alleviated by the antioxidant capacity of the active substance phloretin. Front Cell Dev Biol 2020;8:541260. 33042989 10.3389/fcell.2020.541260 PMC7516399

[bib20] Lian D , Chen MM, Wu H et al. The role of oxidative stress in skeletal muscle myogenesis and muscle dis-ease. Antioxidants 2022;11:755. 35453440 10.3390/antiox11040755 PMC9026549

[bib21] Liang C , Curry BJ, Brown PL et al. Leucine modulates mitochondrial biogenesis and SIRT1-AMPK signaling in C2C12 myotubes. *J Nutr Metab* 2014;2014:1-11. 10.1155/2014/239750 PMC4220583 25400942

[bib22] Lynch CJ , Adams SH. Branched-chain amino acids in metabolic signaling and insulin resistance. Nat Rev Endocrinol 2014;10:723-36. 25287287 10.1038/nrendo.2014.171 PMC4424797

[bib23] Mann G , Mora S, Madu G et al. Branched-chain amino acids: catabolism in skeletal muscle and implications for muscle and whole-body metabolism. Front Physiol 2021;12:702826. 34354601 10.3389/fphys.2021.702826 PMC8329528

[bib24] Mao H , Zhang Y, Ji W et al. Leucine protects bovine intestinal epithelial cells from hydrogen peroxide-induced apoptosis by alleviating oxidative damage. J Sci Food Agric 2022;102:5903-12. 35437753 10.1002/jsfa.11941

[bib25] Mattick JSA , Kamisoglu K, Ierapetritou MG et al. Branched-chain amino acid supplementation: impact on signaling and relevance to critical illness. WIREs Mech Dis 2013;5:449-60. 10.1002/wsbm.1219 PMC4482218 23554299

[bib26] Mizugaki A , Kato H, Takeda T et al. Cystine reduces mitochondrial dysfunction in C2C12 myotubes under moderate oxidative stress induced by H2O2. Amino Acids 2022;54:1203-13. 35715620 10.1007/s00726-022-03176-y PMC9365738

[bib27] Nie C , He T, Zhang W et al. Branched chain amino acids: beyond nutrition metabolism. Int J Mol Sci 2018;19:954. 10.3390/ijms19040954 PMC5979320 29570613

[bib28] Pan R , Chen Y. Management of oxidative stress: crosstalk between brown/beige adipose tissues and skeletal muscles. Front Physiol 2021;12:712372. 34603076 10.3389/fphys.2021.712372 PMC8481590

[bib29] Patyal P , Nguyen B, Zhang X et al. Rho/SRF inhibitor modulates mitochondrial functions. Int J Mol Sci 2022;23:11536. 36232837 10.3390/ijms231911536 PMC9570101

[bib31] Raiteri T , Zaggia I, Reano S et al. The atrophic effect of 1,25(OH)2 Vitamin D3 (Calcitriol) on C2C12 myotubes depends on oxidative stress. Antioxidants 2021;10:1980. 34943083 10.3390/antiox10121980 PMC8750283

[bib32] Ren Y , Li Y, Yan J et al. Adiponectin modulates oxidative stress-induced mitophagy and protects C2C12 myoblasts against apoptosis. Sci Rep 2017;7:3209. 28600493 10.1038/s41598-017-03319-2 PMC5466641

[bib33] Rivera ME , Lyon ES, Johnson MA et al. Leucine increases mitochondrial metabolism and lipid content without altering insulin signaling in myotubes. Biochimie 2020;168:124-33. 31682874 10.1016/j.biochi.2019.10.017

[bib34] Rivera ME , Lyon ES, Johnson MA et al. Effect of valine on myotube insulin sensitivity and metabolism with and without insulin resistance. Mol Cell Biochem 2020;468:169-83. 32222880 10.1007/s11010-020-03720-y

[bib35] Rocha M , Rovira-Llopis S, Banuls C et al. Mitochondrial dysfunction and oxidative stress in insulin resistance. CPD 2013;19:5730-41. 10.2174/13816128113199990373 23448492

[bib36] Rogers SC , Zhang X, Azhar G et al. Exposure to high or low glucose levels accelerates the appearance of markers of endothelial cell senescence and induces dysregulation of nitric oxide synthase. J Gerontol Ser A 2013;68:1469-81. 10.1093/gerona/glt033 PMC3814242 23585419

[bib37] Ruocco C , Segala A, Valerio A et al. Essential amino acid formulations to prevent mitochondrial dysfunction and oxidative stress. Curr Opin Clin Nutr Metab Care 2021;24:88-95. 33060458 10.1097/MCO.0000000000000704

[bib38] Savage DB , Petersen KF, Shulman GI. Disordered lipid metabolism and the pathogenesis of insulin resistance. Physiol Rev 2007;87:507-20. 17429039 10.1152/physrev.00024.2006 PMC2995548

[bib39] Schnuck JK , Sunderland KL, Gannon NP et al. Leucine stimulates PPARβ/δ-dependent Mi-tochondrial biogenesis and oxidative metabolism with enhanced GLUT4 content and glucose uptake in myotubes. Biochimie 2016;128-129: 1–7. 10.1016/j.biochi.2016.06.009 27345255

[bib40] Tamanna N , Mahmood N. Emerging roles of branched-chain amino acid supplementation in human diseases. Int Sch Res Notices 2014;2014:1-8. 10.1155/2014/235619 PMC4897441 27351005

[bib41] Thandapani P , Kloetgen A, Witkowski MT et al. Valine TRNA levels and availability regulate complex I assembly in leukaemia. Nature 2022;601:428-33. 34937946 10.1038/s41586-021-04244-1 PMC9116157

[bib42] Tom A , Nair KS. Assessment of branched-chain amino acid status and potential for biomarkers. J Nutr 2006;136:324S-30S. 16365107 10.1093/jn/136.1.324S

[bib43] Valerio A , D'Antona G, Nisoli E. Branched-chain amino acids, mitochondrial biogenesis, and healthspan: an evolutionary perspective. Aging 2011;3:464-78. 21566257 10.18632/aging.100322 PMC3156598

[bib44] Vanweert F , Schrauwen P, Phielix E. Role of branched-chain amino acid metabolism in the pathogenesis of obesity and Type 2 diabetes-related metabolic disturbances BCAA metabolism in Type 2 diabetes. Nutr Diabetes 2022;12:35. 35931683 10.1038/s41387-022-00213-3 PMC9356071

[bib45] Verma A , Azhar G, Zhang X et al. P. gingivalis-LPS induces mitochondrial dysfunction mediated by neuroinflammation through oxidative stress. Int J Mol Sci 2023;24:950. 36674463 10.3390/ijms24020950 PMC9861869

[bib46] Viana LR , Tobar N, Busanello ENB et al. Leucine-rich diet induces a shift in tumour metabolism from glycolytic towards oxidative phosphorylation, reducing glucose consumption and metastasis in Walker-256 tumour-bearing rats. Sci Rep 2019;9:15529. 31664147 10.1038/s41598-019-52112-w PMC6820796

[bib47] Walters JW , Amos D, Ray K et al. Mitochondrial redox status as a target for cardiovascular disease. Curr Opin Pharmacol 2016;27:50-5. 26894468 10.1016/j.coph.2016.01.006 PMC4808324

[bib48] Wolfe RR. Branched-chain amino acids and muscle protein synthesis in humans: myth or reality? *J Int Soc Sports Nutr* 2017;14:30. 10.1186/s12970-017-0184-9 PMC5568273 28852372

[bib49] Wu S , Liu X, Cheng L et al. Protective mechanism of leucine and isoleucine against H2O2-induced oxidative damage in bovine mammary epithelial cells. Oxid Med Cell Long 2022;2022:1-22. 10.1155/2022/4013575 PMC8964234 35360198

[bib50] Ye Z , Wang S, Zhang C et al. Coordinated modulation of energy metabolism and inflammation by branched-chain amino acids and fatty acids. Front Endocrinol 2020;11:617. 10.3389/fendo.2020.00617 PMC7506139 33013697

[bib51] Yoneshiro T , Kataoka N, Walejko JM et al. Metabolic flexibility via mitochondrial BCAA carrier SLC25A44 is required for optimal fever. Cell Bio 2021;10:e66865. 10.7554/eLife.66865 PMC8137140 33944778

[bib52] Yoon MS. The emerging role of branched-chain amino acids in insulin resistance and metabolism. Nutrients 2016;8:405. 27376324 10.3390/nu8070405 PMC4963881

[bib53] Zhang X , Azhar G, Nagano K et al. Differential vulnerability to oxidative stress in rat cardiac myocytes versus fibroblasts. J Am Coll Cardiol 2001;38:2055-62. 11738315 10.1016/s0735-1097(01)01665-5

[bib54] Zhang X , Azhar G, Wei JY. The expression of MicroRNA and MicroRNA clusters in the aging heart. PLoS One 2012;7:e34688. 22529925 10.1371/journal.pone.0034688 PMC3329493

[bib55] Zhao MJ , Yuan S, Zi H et al. Oxidative stress links aging-associated cardiovascular diseases and prostatic diseases. Oxid Med Cell Long 2021;2021:1-12. 10.1155/2021/5896136 PMC8313344 34336107

[bib56] Zhao R , Jiang S, Zhang L et al. Mitochondrial electron transport chain, ROS generation and uncoupling (review). Int J Mol Med 2019;44:3-15. 31115493 10.3892/ijmm.2019.4188 PMC6559295

[bib57] Zheng J. Energy metabolism of cancer: glycolysis versus oxidative phosphorylation (review). Onco Letters 2012;4:1151-7. 10.3892/ol.2012.928 PMC3506713 23226794

