# Supplementary material for: Valine improves mitochondrial function and protects against oxidative stress
Source: Biosci Biotechnol Biochem. 2023 Dec 13;88(2):168–76. doi: 10.1093/bbb/zbad169 (PMC10807754; doi:10.1093/bbb/zbad169)
Supplement: zbad169_Supplemental_File [file zbad169_supplemental_file.docx]

**Supplementary Data**

**Supplementary Fig. 1:** Effect of valine treatment on cells viability with MTS assay. C2C12 cells were treated with 1.0, 1.5 mM concentrations of valine for 24 hours and untreated cells were used as control.

**Supplementary Fig. 2:** Effect of valine treatment on ATP and NAD/ NADH production. (A) ATP and (B) NAD/ NADH production levels were analyzed. * *p* < 0.05, **** *p*< 0.0001 (*n=3)*.

**Supplementary Fig. 3:** Effect of H_2_O_2_ on cells viability. C2C12 cells were treated with different concentrations of H_2_O_2_ for 6 hours and untreated cells were used as control (n=3).

**Supplementary Fig. 4:** Effect of different doses of H_2_O_2_ on OCR after Valine treatment.

**Supplementary Fig. 5:** Valine maintained cellular viability after H_2_O_2_ treatment. The cellular viability of C2C12 cells was reduced by H_2_O_2_ treatment, which was significantly recovered after valine addition *(n=3).* *****p* < 0.0001.

**Supplement Table S1.** List of primer sequences used for qPCR analysis.

| Primer Name Primer Sequence (5’-3’) |
| --- |
| PGC-1α-F 5'- ACCCACAGAGAACAGAAACAG-3'  PGC-1α-R 5'- GGGTCAGAGGAAGAGATAAAGTTG-3'  PGC-1β –F 5'- ACTACTTCGCTGACACGCAG-3'  PGC-1β –R 5'-CTCTGAGTTCTCTGGGCACC-3'  MFN1-F 5'- TGGCATCTGTGGCCGAGTT-3'  MFN1-R 5'-GAAACAGGTTCTGCCATTATGCT -3'  MFN2-F 5'- CGCGCTTATCCACTTCCCTC-3'  MFN2-R 5'- AGAAGAGCAGGGACATTGCG-3'  Fis1-F 5'- TGACATCCGTAAAGGCATCG-3'  Fis1-R 5'-CTTCTCGTATTCCTTGAGCCG -3'  Opa1-F 5'- GGAGAACCATATTCGTTTTGACC-3'  Opa1-R 5'- AGAGCTGTTCCCTTTTCCTG-3' |
